# Supplementary figures and images for: Ferrostatin-1 post-treatment attenuates acute kidney injury in mice by inhibiting ferritin production and regulating iron uptake-related proteins
Source: PeerJ. 2023 Sep 8;11:e15786. doi: 10.7717/peerj.15786 (PMC10494833; doi:10.7717/peerj.15786)

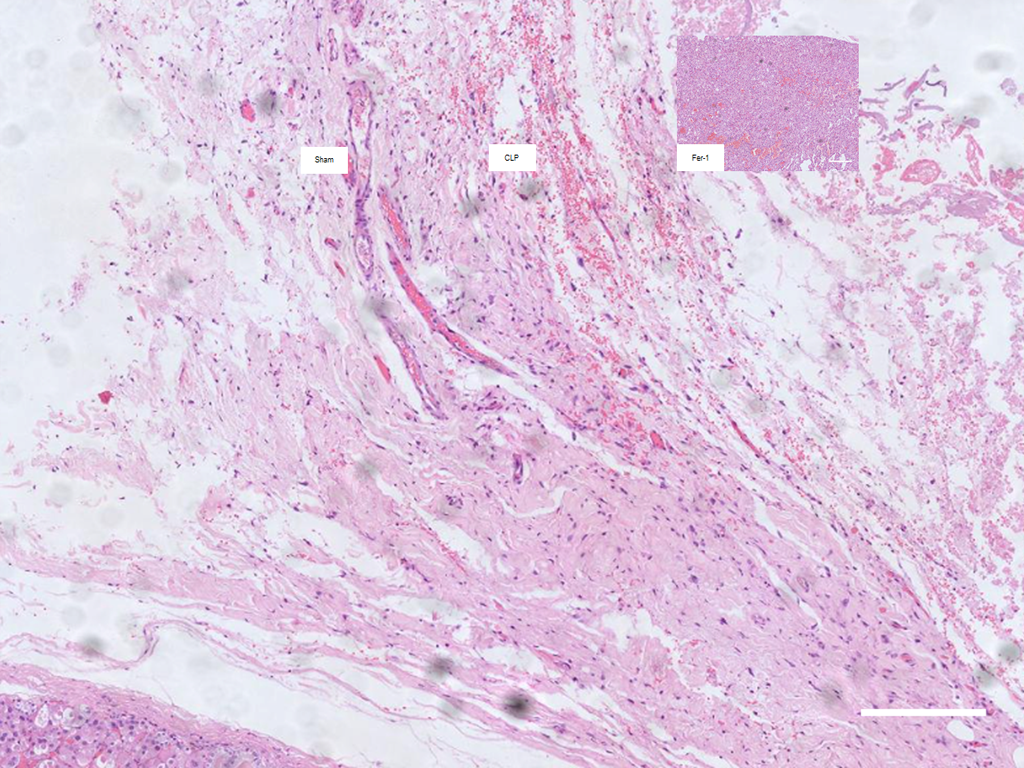

Supplement: Supplemental Information 1 [file peerj-11-15786-s001.zip › figure1/images/1A-CLP.tif]

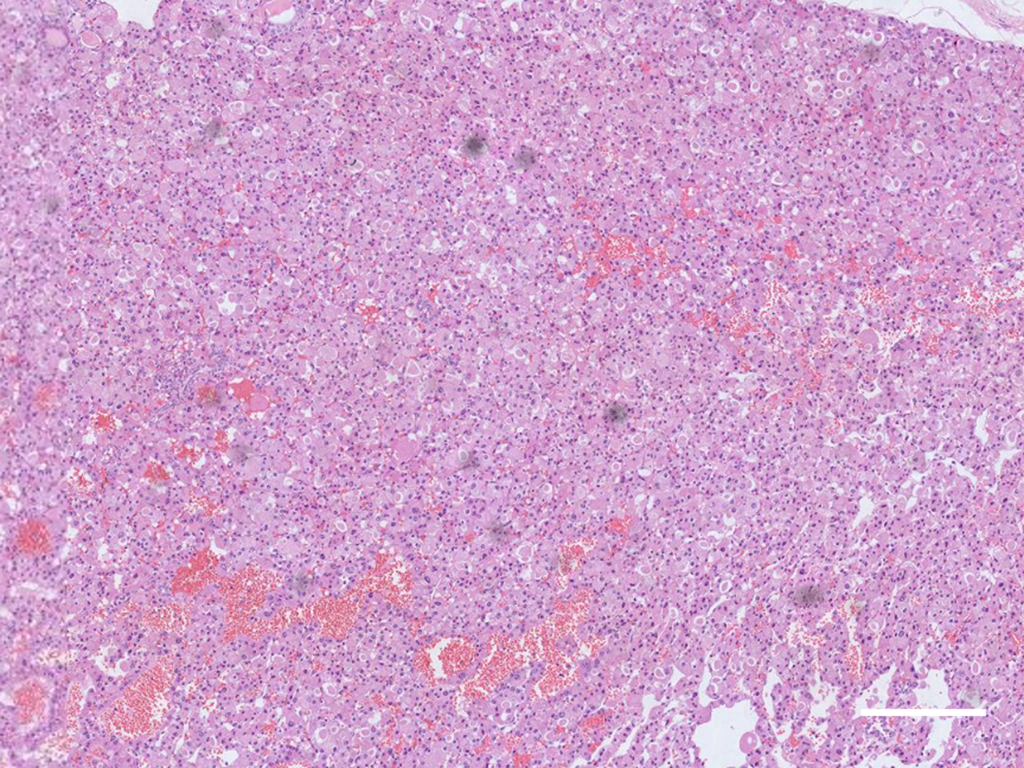

Supplement: Supplemental Information 1 [file peerj-11-15786-s001.zip › figure1/images/1A-Fer-1.tif]

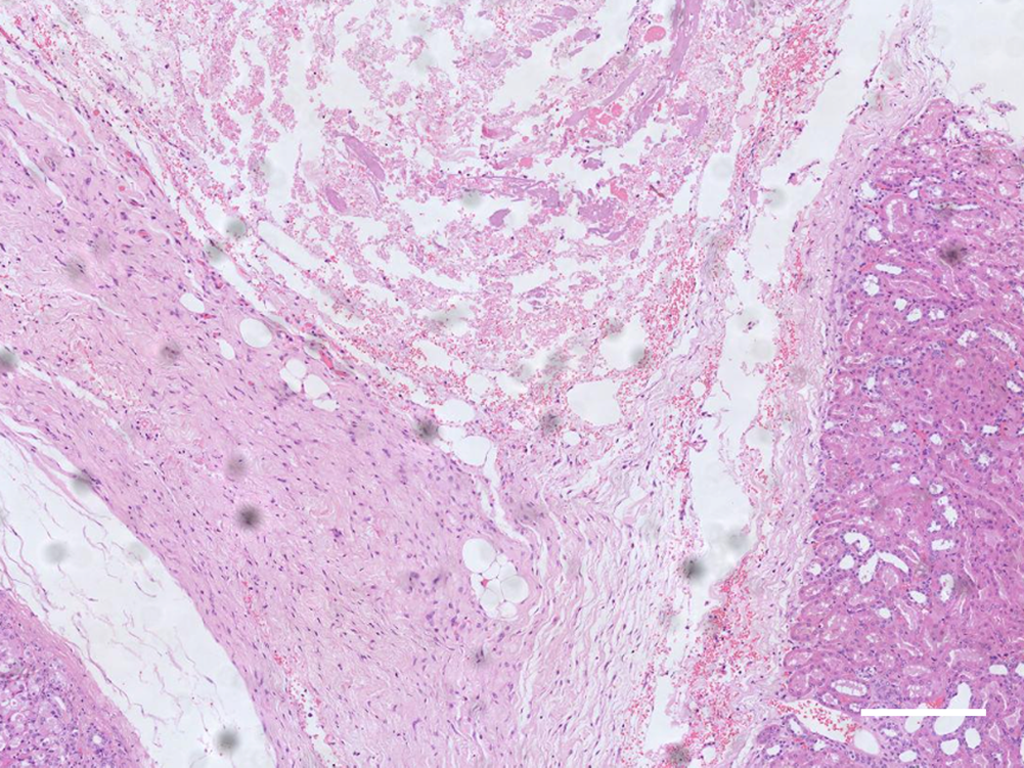

Supplement: Supplemental Information 1 [file peerj-11-15786-s001.zip › figure1/images/1A-Sham.tif]

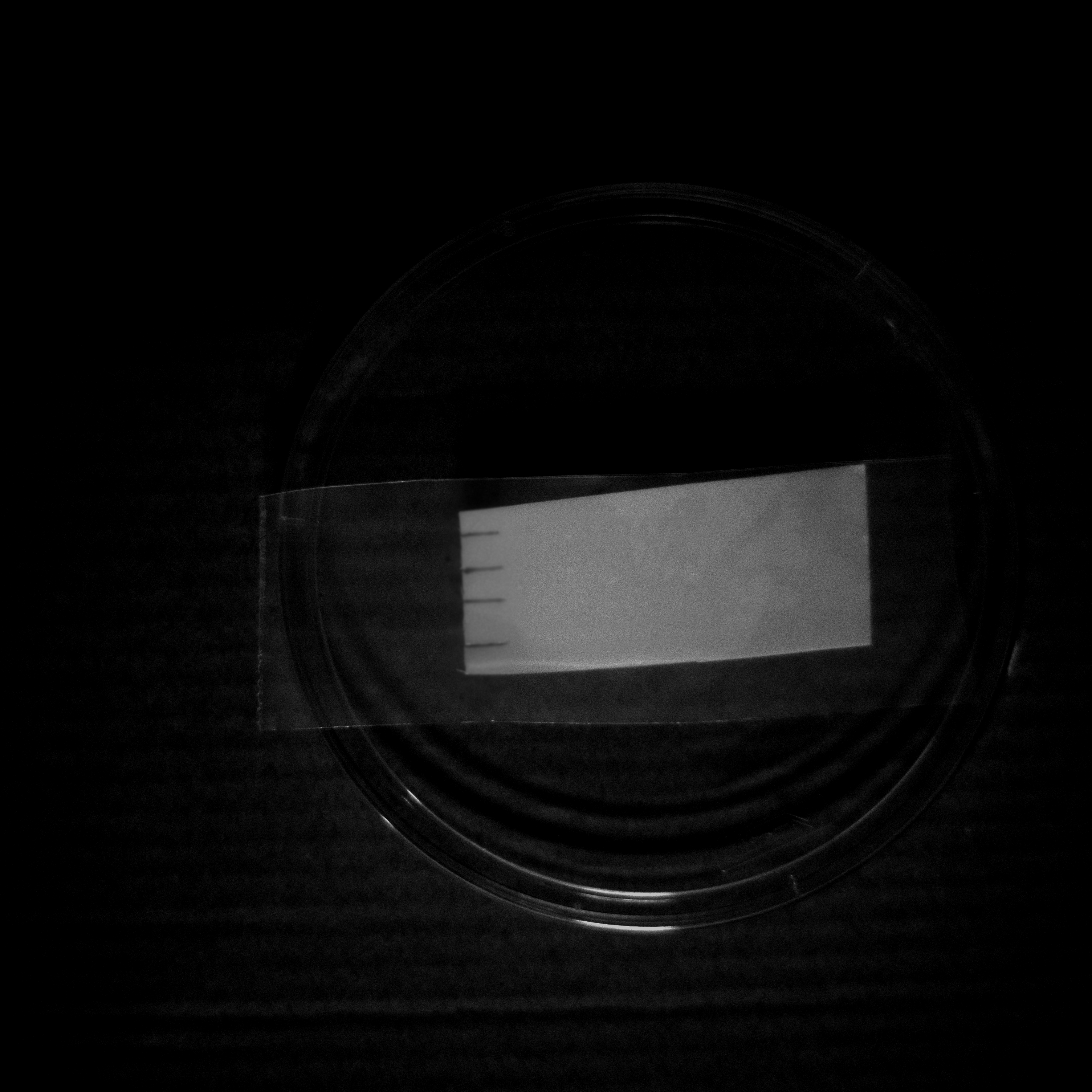

Supplement: Supplemental Information 2 [file peerj-11-15786-s002.zip › figure2/images/2E FTH1+NRF2 BF.png]

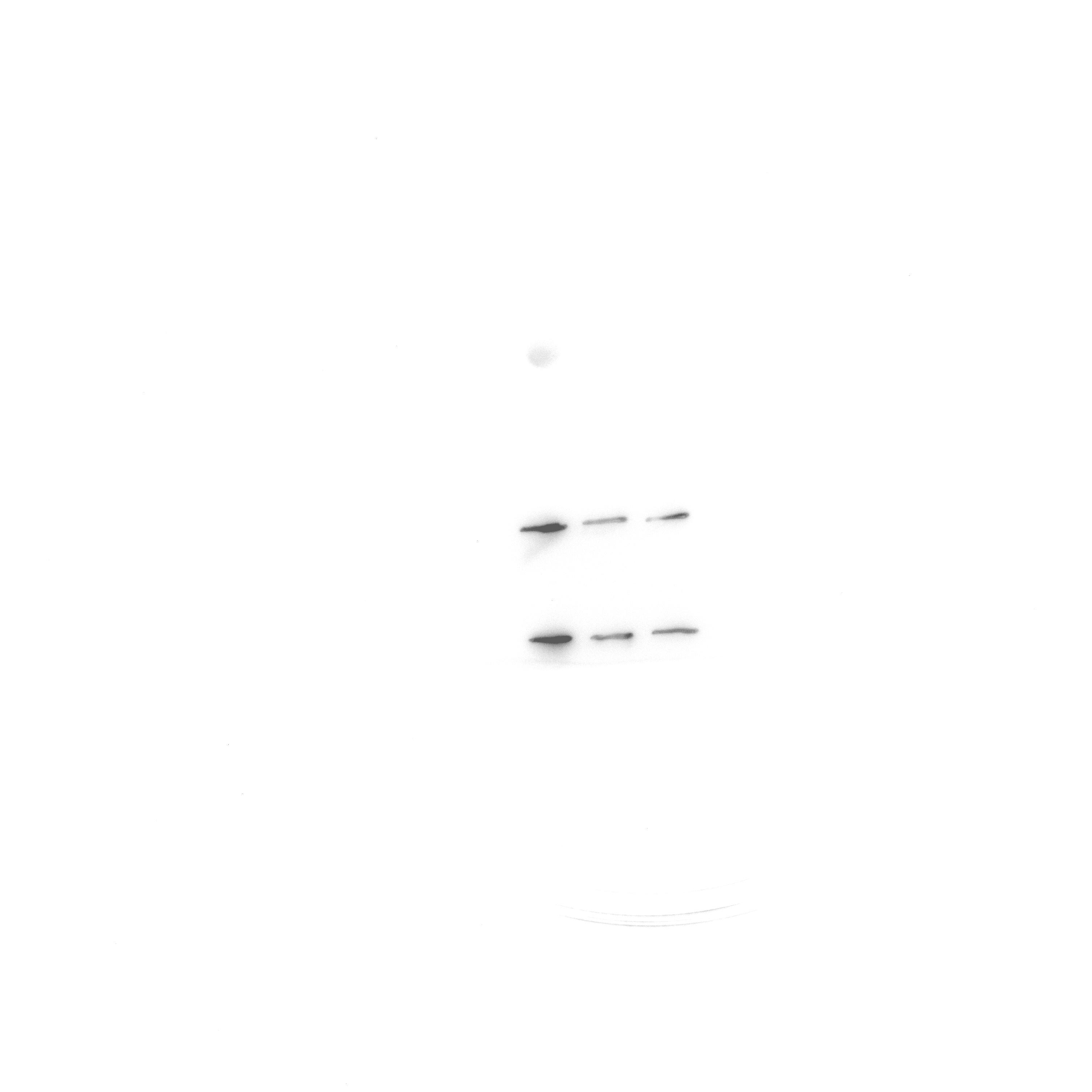

Supplement: Supplemental Information 2 [file peerj-11-15786-s002.zip › figure2/images/2E FTH1+NRF2.png]

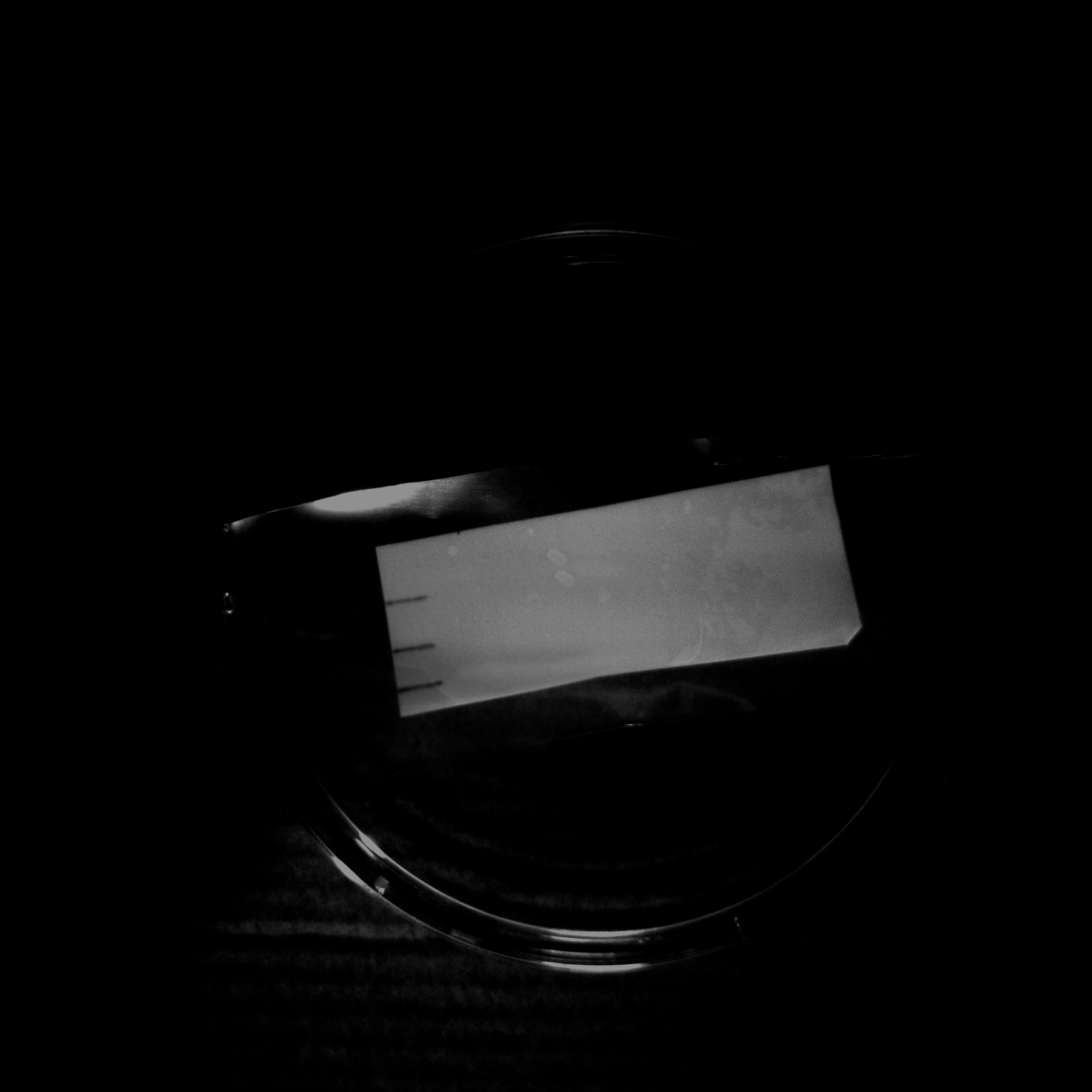

Supplement: Supplemental Information 2 [file peerj-11-15786-s002.zip › figure2/images/2E GPX4+SLAC7A11 BF.png]

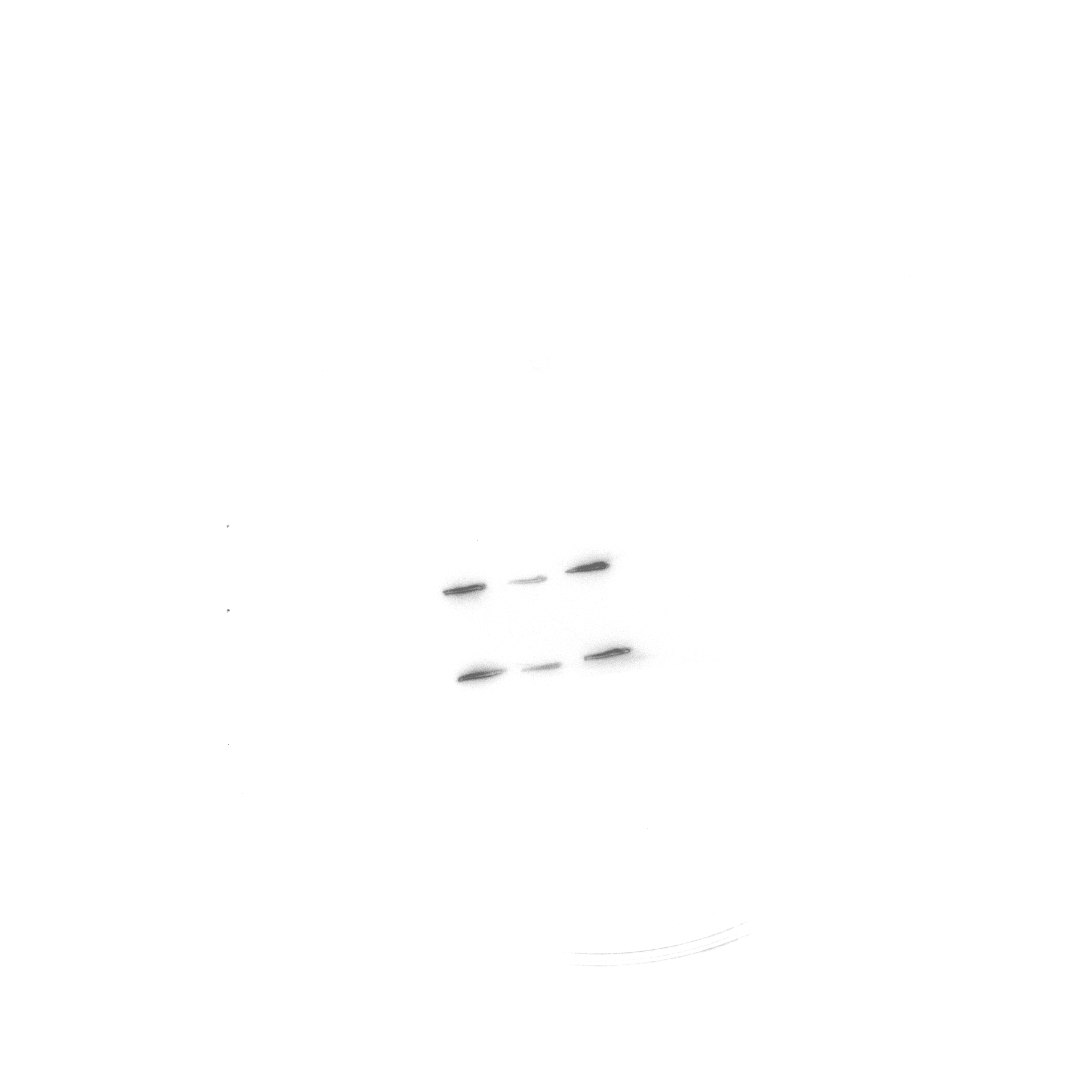

Supplement: Supplemental Information 2 [file peerj-11-15786-s002.zip › figure2/images/2E GPX4+SLAC7A11.png]

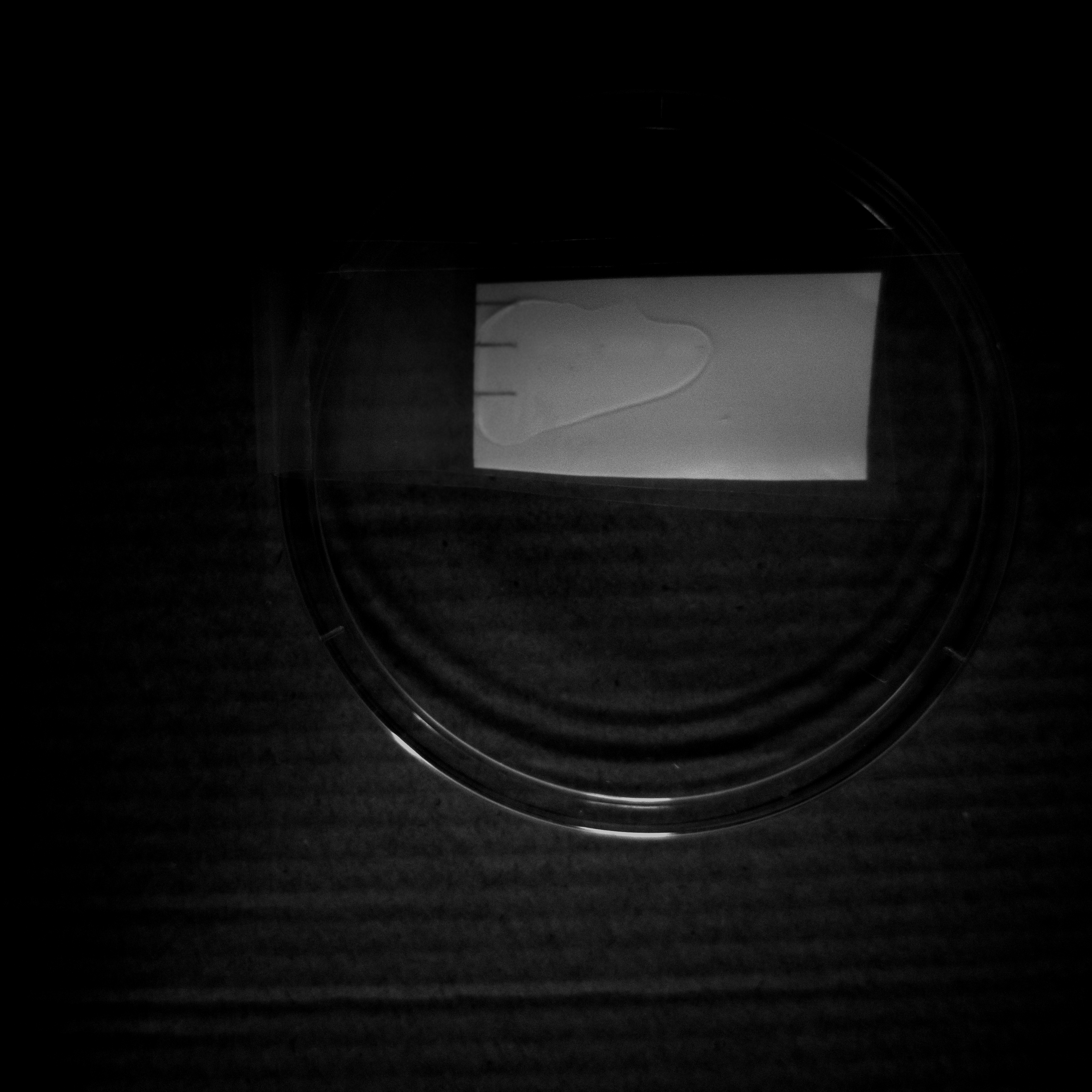

Supplement: Supplemental Information 2 [file peerj-11-15786-s002.zip › figure2/images/2E β-Actin BF.png]

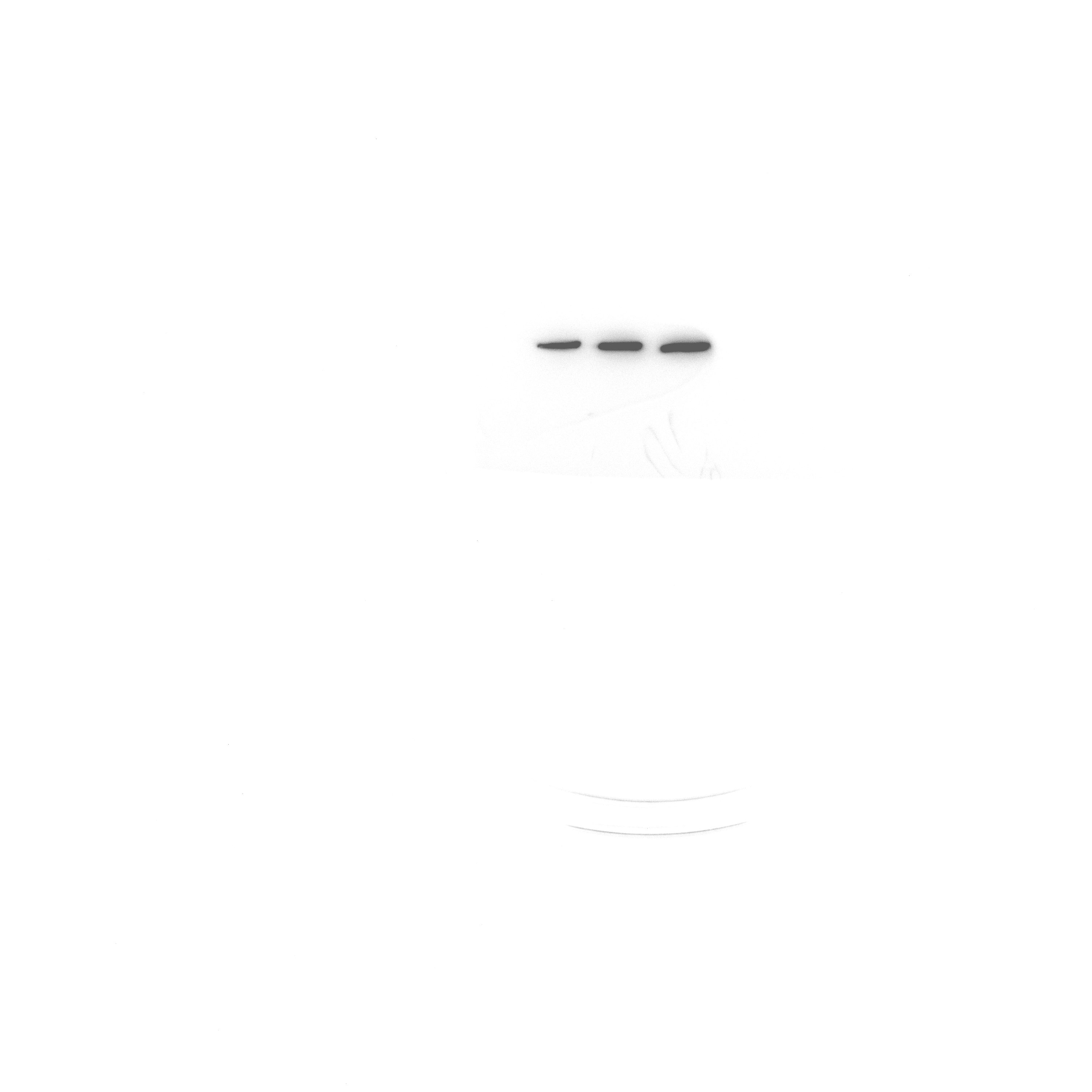

Supplement: Supplemental Information 2 [file peerj-11-15786-s002.zip › figure2/images/2E β-Actin.png]

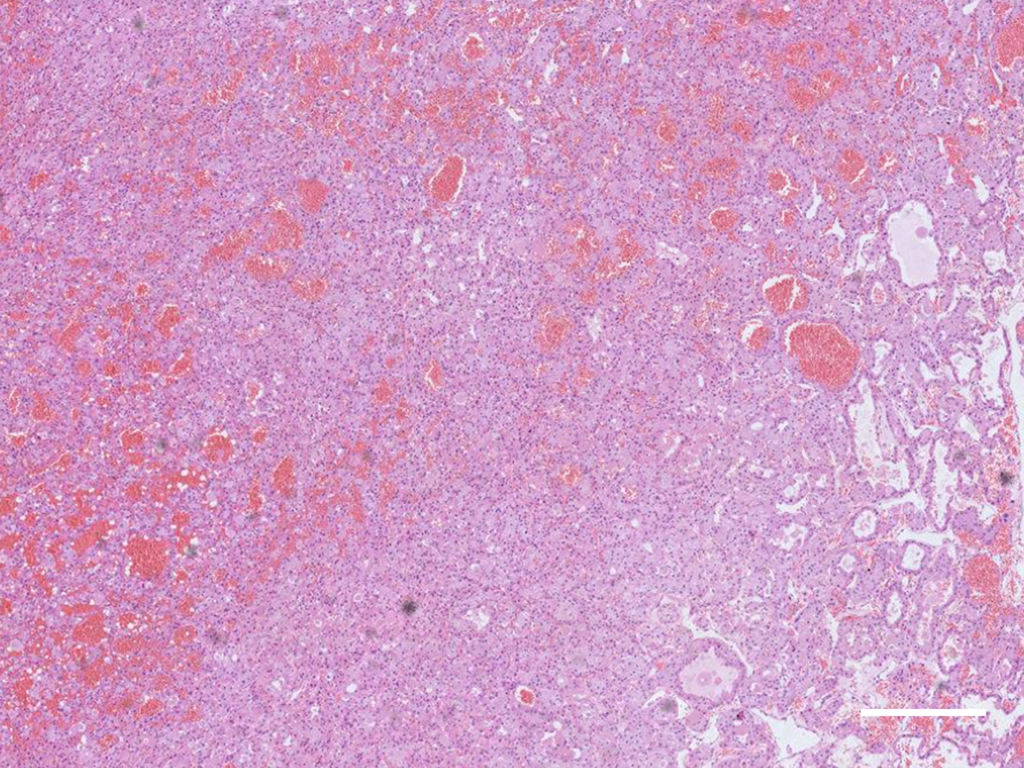

Supplement: Supplemental Information 3 [file peerj-11-15786-s003.zip › figure3/images/3A-Con.tif]

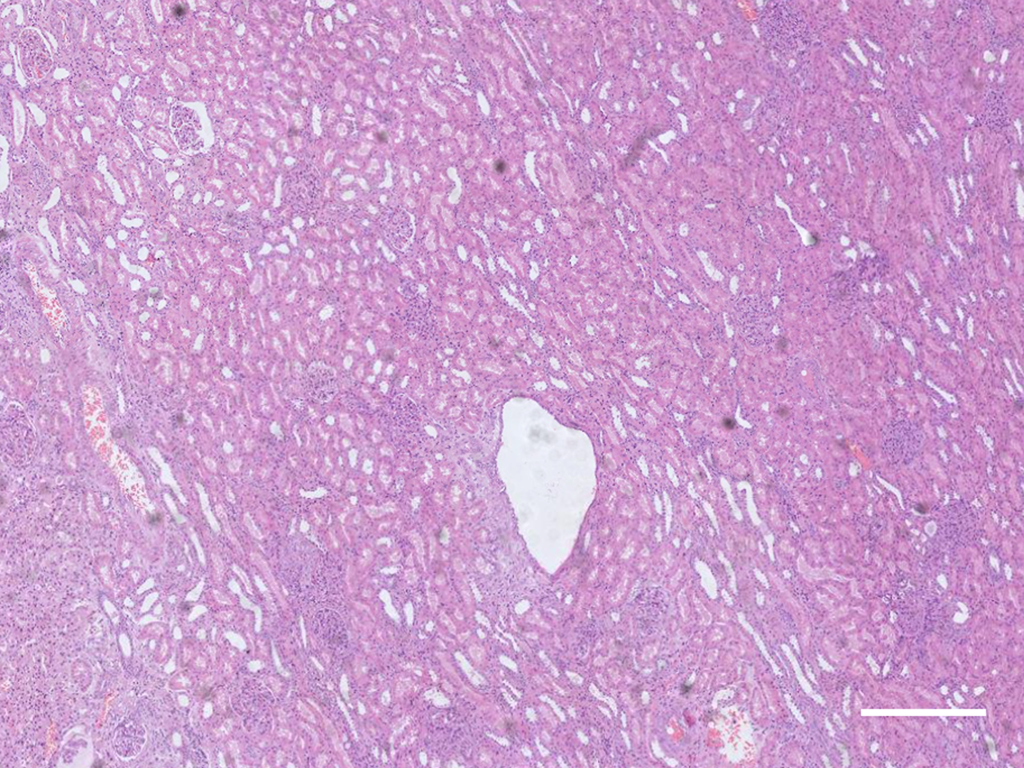

Supplement: Supplemental Information 3 [file peerj-11-15786-s003.zip › figure3/images/3A-Fer-1.tif]

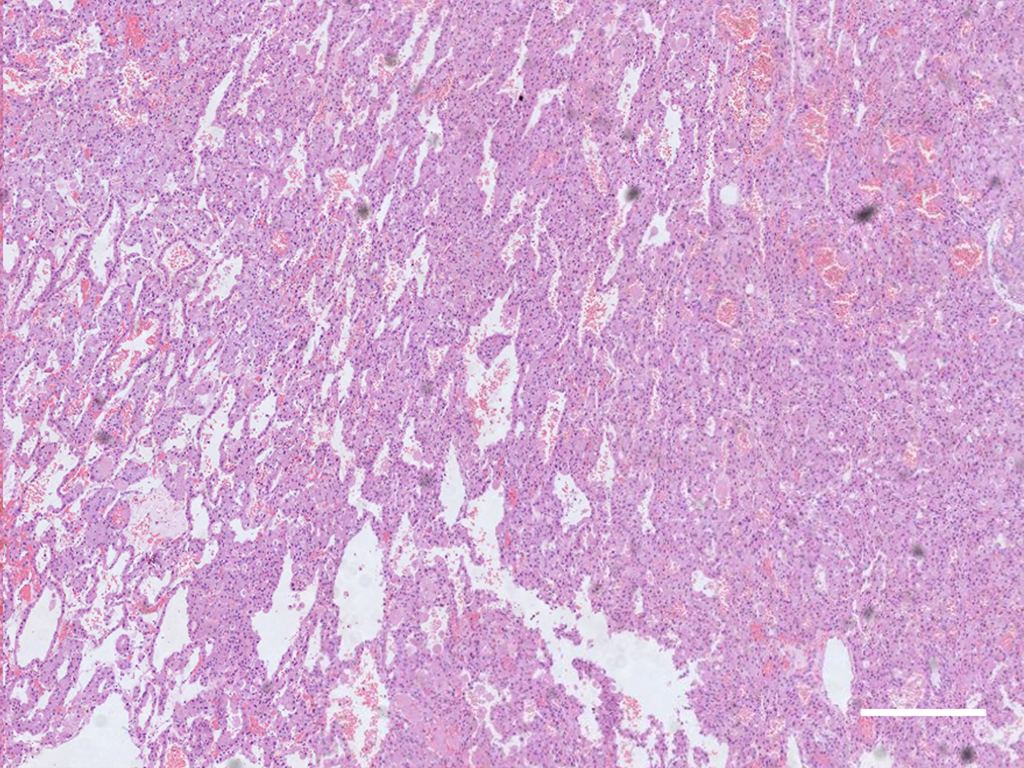

Supplement: Supplemental Information 3 [file peerj-11-15786-s003.zip › figure3/images/3A-LPS.tif]

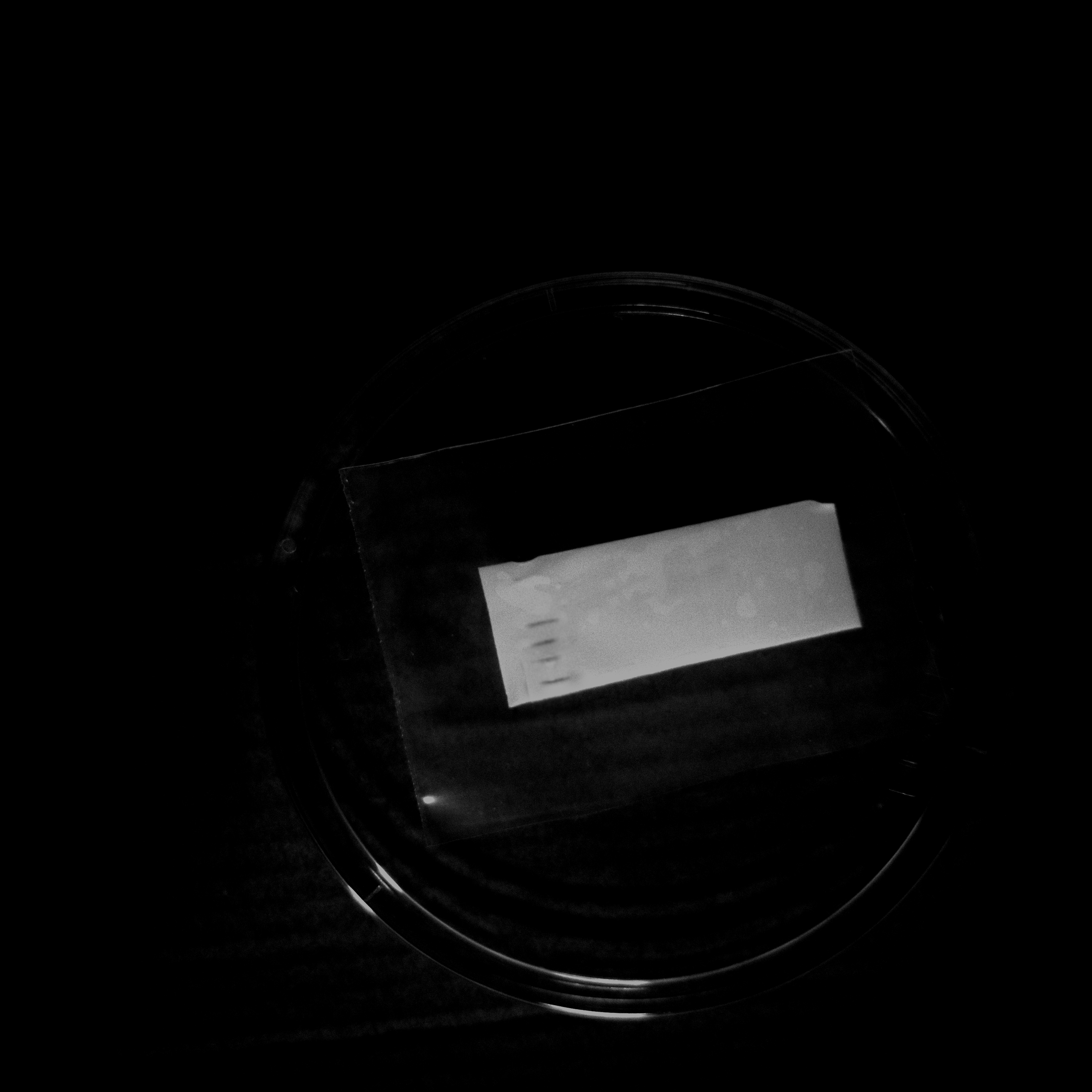

Supplement: Supplemental Information 3 [file peerj-11-15786-s003.zip › figure3/images/3G FTH1+NRF2 BF.png]

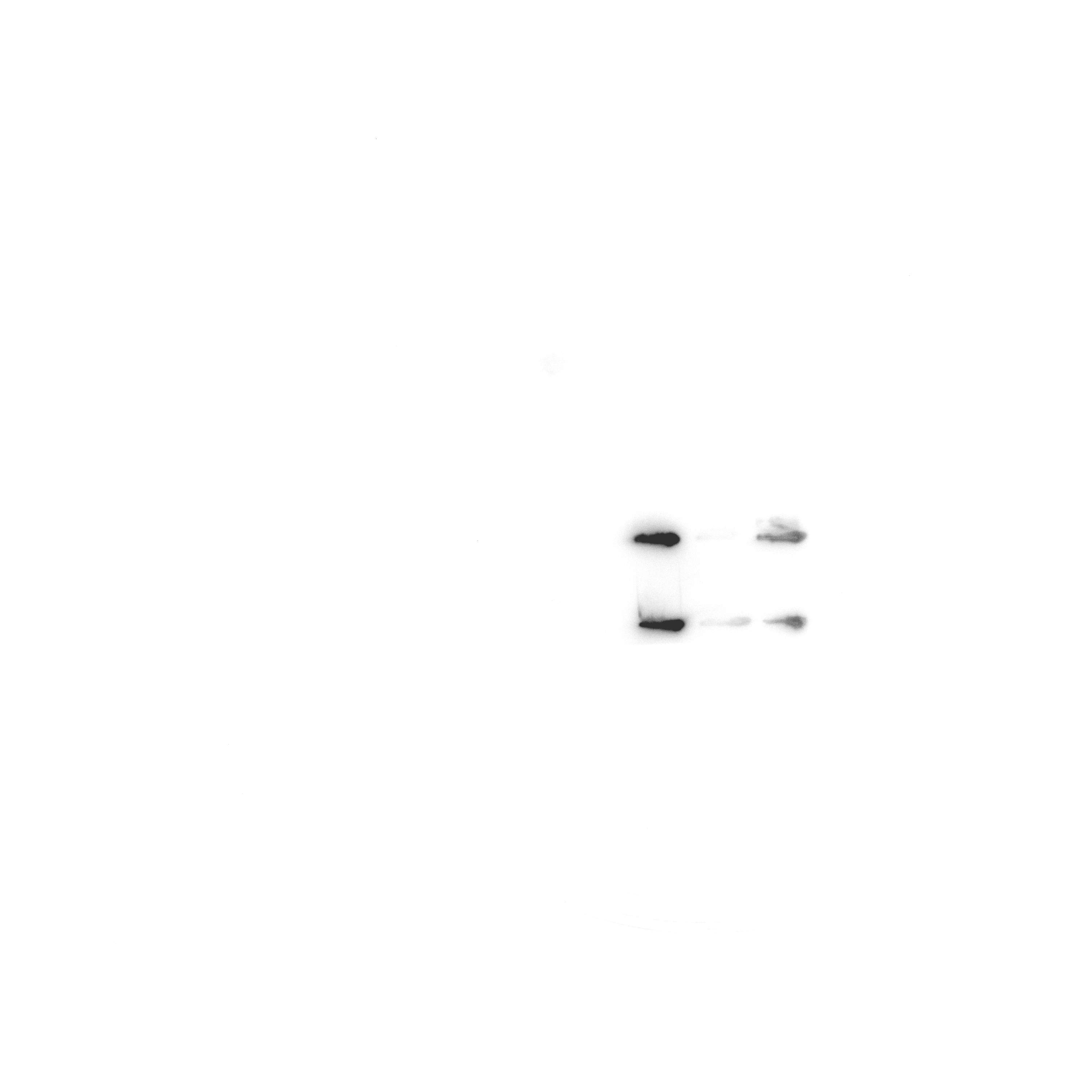

Supplement: Supplemental Information 3 [file peerj-11-15786-s003.zip › figure3/images/3G FTH1+NRF2.png]

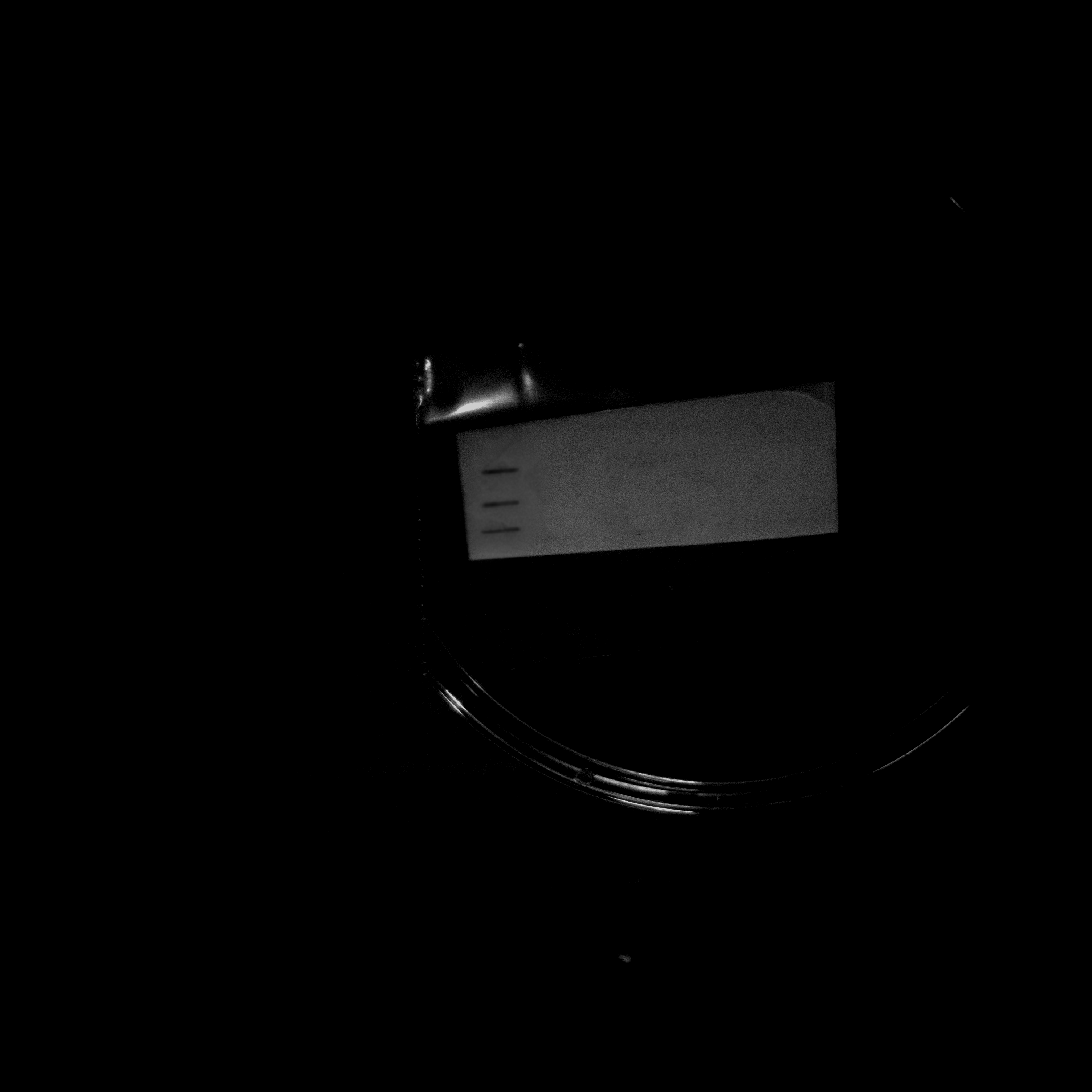

Supplement: Supplemental Information 3 [file peerj-11-15786-s003.zip › figure3/images/3G GPX4+SLC7A11 BF.png]

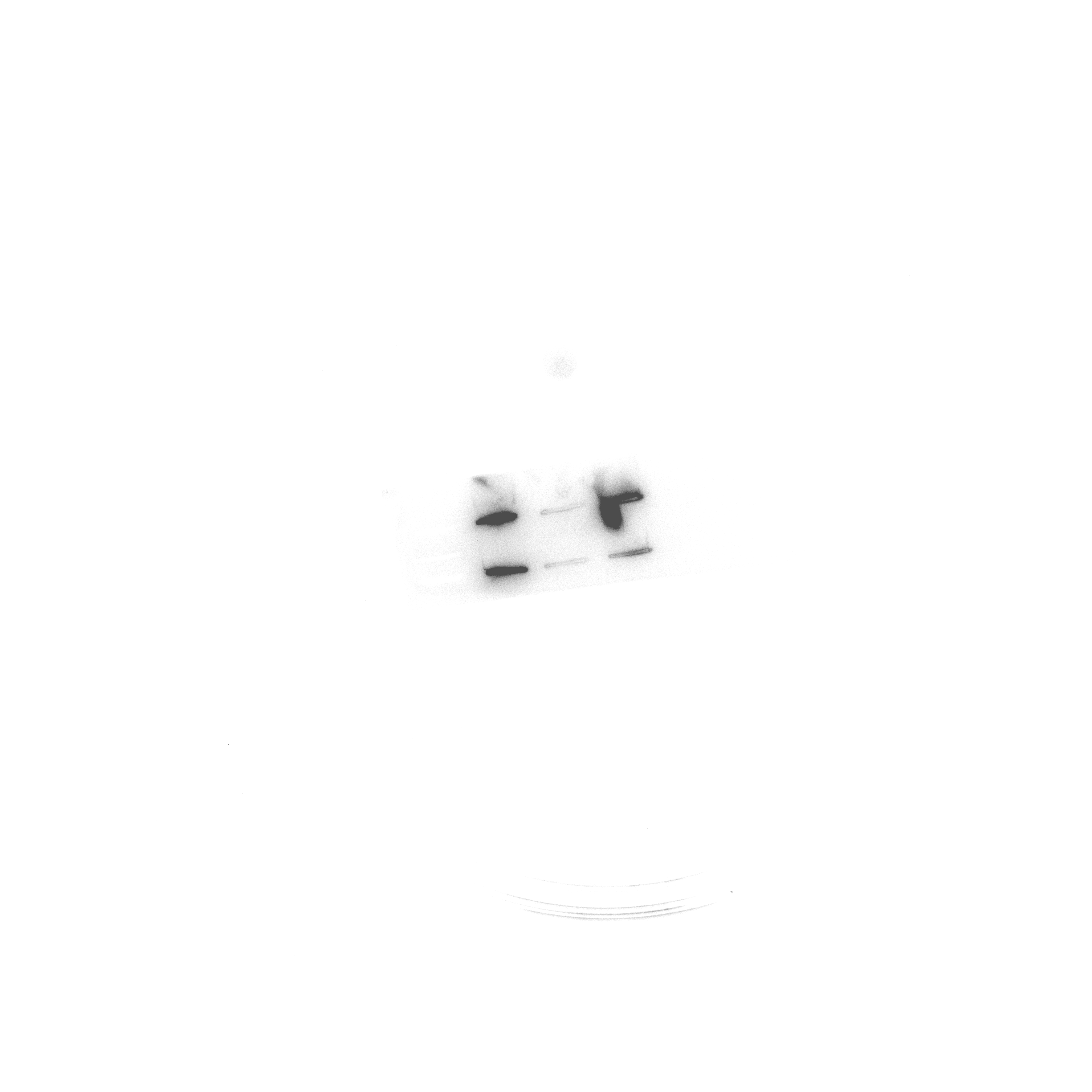

Supplement: Supplemental Information 3 [file peerj-11-15786-s003.zip › figure3/images/3G GPX4+SLC7A11.png]

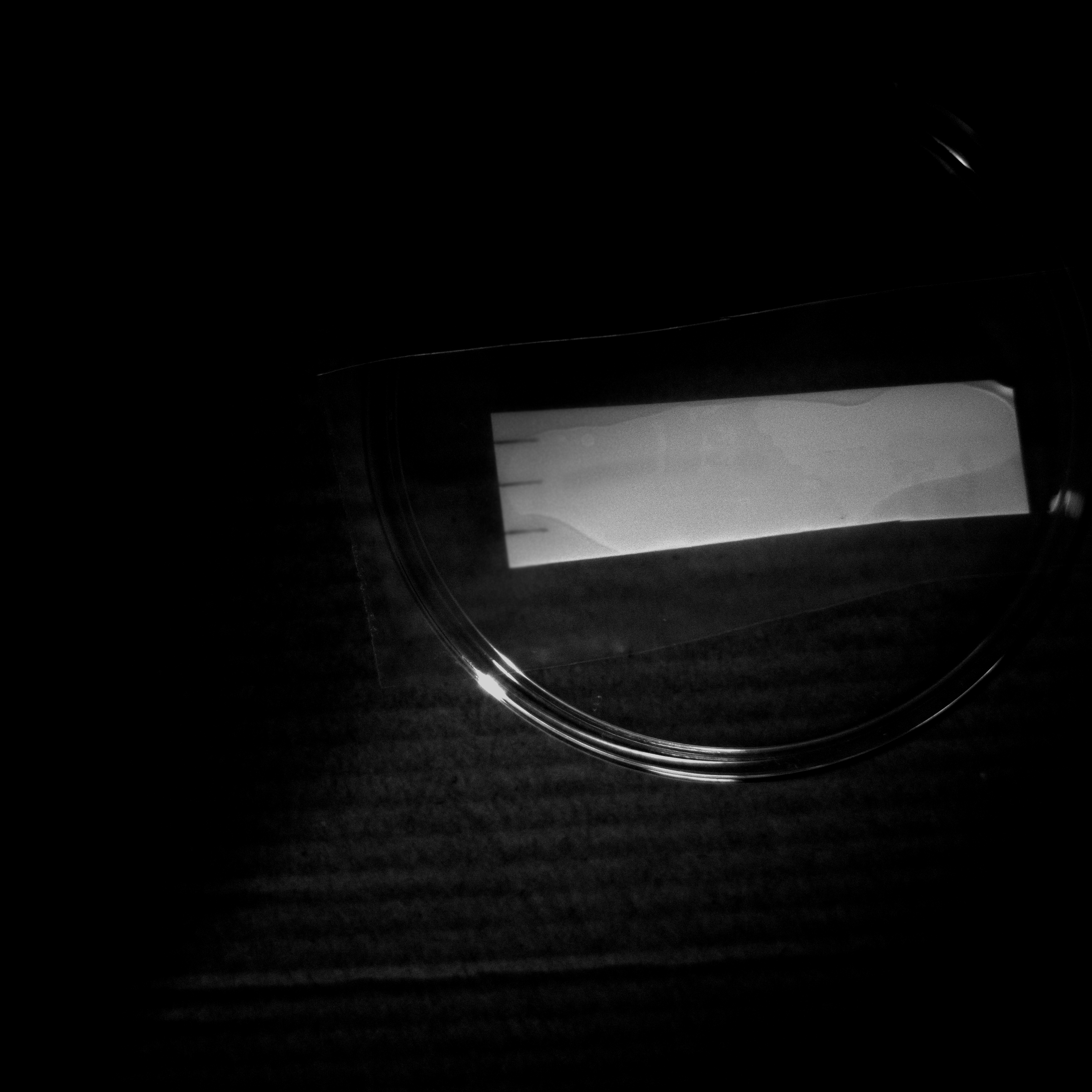

Supplement: Supplemental Information 3 [file peerj-11-15786-s003.zip › figure3/images/3G β-Actin BF.png]

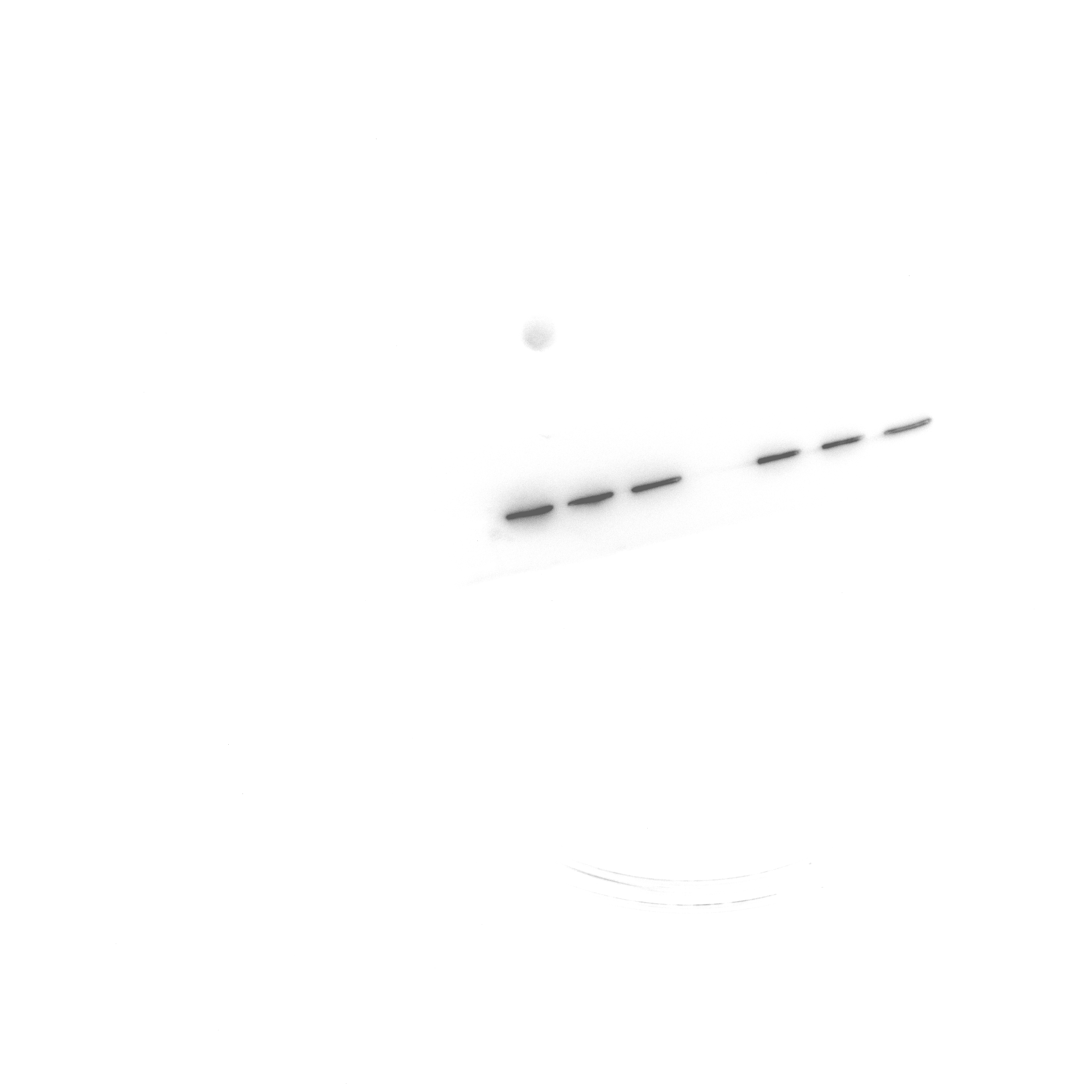

Supplement: Supplemental Information 3 [file peerj-11-15786-s003.zip › figure3/images/3G β-Actin.png]
